# Synergistic and antagonistic drug interactions are prevalent but not conserved across acute myeloid leukemia cell lines

**DOI:** 10.21203/rs.3.rs-4159724/v1

**Published:** 2024-08-26

**Authors:** Fatma Neslihan Kalkan, Muhammed Sadik Yildiz, N. Ezgi Wood, Michael Farid, Melissa McCoy, Milo Lin, Chengcheng Zhang, Bruce Posner, Stephen S. Chung, Erdal Toprak

**Affiliations:** 1 Department of Internal Medicine, University of Texas Southwestern Medical Center, Dallas, TX; 2 Department of Pharmacology, University of Texas Southwestern Medical Center, Dallas, TX; 3 Department of Biochemistry, University of Texas Southwestern Medical Center, Dallas, TX; 4 Lyda Hill Department of Bioinformatics, University of Texas Southwestern Medical Center, Dallas, TX; 5 Department of Physiology, University of Texas Southwestern Medical Center, Dallas, TX

## Abstract

Acute myeloid leukemia (AML) is the most prevalent type of leukemia in adults. Its heterogeneity, both between patients and within the same patient, is often a factor contributing to poor treatment outcomes. Despite advancements in AML biology and medicine in general, the standard AML treatment, the combination of cytarabine and daunorubicin, has remained the same for decades. Combination drug therapies are proven effective in achieving targeted efficacy while minimizing drug dosage and unintended side effects, a common problem for older AML patients. However, a systematic survey of the synergistic potential of drug-drug interactions in the context of AML pathology is lacking. Here, we examine the interactions between 15 commonly used cancer drugs across distinct AML cell lines and demonstrate that synergistic and antagonistic drug-drug interactions are widespread but not conserved across these cell lines. Notably, enasidenib and venetoclax, recently approved anticancer agents, exhibited the highest counts of synergistic interactions and the fewest antagonistic ones. In contrast, 6-Thioguanine, a purine analog, was involved in the highest number of antagonistic interactions. The interactions we report here cannot be attributed solely to the inherent natures of these three drugs, as each drug we examined was involved in several synergistic or antagonistic interactions in the cell lines we tested. Importantly, these drug-drug interactions are not conserved across cell lines, suggesting that the success of combination therapies might vary significantly depending on AML genotypes. For instance, we found that a single mutation in the TF1 cell line could dramatically alter drug-drug interactions, even turning synergistic interactions into antagonistic ones. Our findings provide a preclinical survey of drug-drug interactions, revealing the complexity of the problem.

## Introduction

Acute myeloid leukemia (AML) is a malignant blood disorder originating from the clonal proliferation of abnormally or incompletely differentiated blood cells of the myeloid lineage^1^. AML is the most common leukemia type in adults^2^, affecting approximately 20,000 people in the United States with an average diagnosis age of 68^3^. Although the treatment outcomes for AML patients younger than 60 are in general better, the 5-year survival rate for patients over 65 is only about 6.9–8.9%^4^. This discrepancy is often attributed to the severe side effects associated with the intensive chemotherapy regimens that AML treatment requires^5^. This often means that aggressive treatment options are entirely off the table for older AML patients, and those with comorbidities^6^. To address this significant clinical problem, there is growing interest in developing new drugs with fewer side effects and using combinations of existing cancer medications for greater efficacy at lower doses to reduce dose-dependent side effects. For the latter, the standard approach is to seek synergistic drug combinations in which combined effects of drugs are higher than the sum of each drug’s inhibitory effect when used alone^7^. However, despite several studies attempting to identify synergistic drug combinations for AML, the traditional treatment regimen for AML with the combined use of cytarabine and daunorubicin, has remained largely unchanged for several decades^8^.

**Figure 1.**
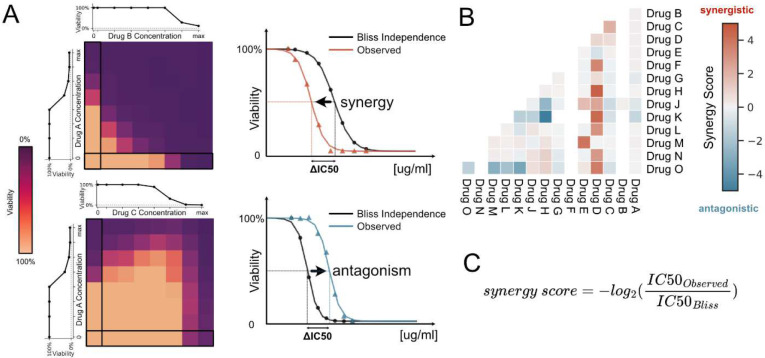
High-throughput drug combination screen. **A.** 105 possible drug pairs of the selected 15 drugs are screened against each other at 8 pre-selected drug concentrations. Two 8×8 heat maps show pairwise drug interactions. Darker tones indicate higher cell death. Drug A (Y-axis) combined with Drug B (X-axis) shows synergy, while Drug A with Drug C shows antagonism. Single drug response curves determine dose ranges. Bold-framed areas represent zero doses. IC50 values on plates demonstrate synergistic (lower than expected) or antagonistic (higher than expected) effects compared to theoretical IC50 ranges. **B.** A two-parameter sigmoid model (see Methods section) fitted for every row and IC_50_ is estimated and compared with the Bliss Independence model. When the observed effect is larger than the predicted effect (red line), drug-drug interaction is considered synergistic. When the observed effect is lower than the predicted effect (blue line), drug-drug interaction is considered antagonistic. **C.** Synergy scores are predicted by calculating the median synergy score (S = −log_2_(ΔIC_50_), Methods) across all measured combination regimes. If the median value is positive, drug-drug interaction is marked as synergistic and the maximum achievable −log_2_(ΔIC_50_) value is used as the synergy score (red pixels). If the median value is negative, drug-drug interaction is marked as antagonistic and the minimum −log_2_(ΔIC_50_) value is used as the synergy score (blue pixels).

AML is a clinically and genetically heterogeneous disease^9^ which complicates the prediction of how an AML patient will respond to the cytarabine and daunorubicin combination, as well as to newly developed drug(s) or drug combination therapies. This problem has become increasingly challenging and relevant with the introduction of novel drugs for AML^10^. Between 2017 and 2019, the Food and Drug Administration (FDA) approved 8 new compounds for treating AML^10^. However, detailed studies assessing these compounds’ interactions with each other, or other drugs, remain limited. Here, to aid clinical studies aiming to design effective combinatorial therapies for AML patients, we conducted an extensive systematic study to measure interactions between 15 drugs that have been used to treat several different types of leukemia in the clinic (see Figure 1, Supplementary Table 4). We tested *in vitro* efficacies of 105 drug pairs (105 = 15 × 14 / 2) against six AML cell lines representing a breadth of AML subtypes (**Supplementary Table 5**). 19 drugs were initially selected for this study; however, hydroxyurea, glasdegib, prednisone, and dexamethasone were excluded due to insufficient killing effect when used alone. Our objectives are to find synergistic drug pairs and to determine whether these drug interactions are conserved across different cell lines.

## Methods:

### Cell growth conditions and High-throughput assay

Cell lines were plated at the following densities in a total volume of 60 uL per well in 384 well microtiter plates (Greiner catalog 781098): HL-60 and K562 cells at 1200 cells/well, Kasumi-1 at 1600 cells/well, TFI-IDH1 mutant at 2500 cells/well and FKH01 at 10,000 cells/well. Cells were incubated overnight at 37 degrees Celsius and 5% CO_2_. The following day, test compounds were dissolved in DMSO or aqueous solvent and added to 384-well plates using an Echo555 or Echo 655 liquid handler (Beckman, Inc). For combination assays, pairs of compounds were dosed in 8×8 grids at concentrations determined by the IC_50_ for each compound. One compound of each pair was dosed in columns and the other in rows. The effect of each compound alone was determined by assaying cell viability in the absence of the other compound (single agent control). All compounds were also tested against themselves to measure additive effects (sham control). DMSO treated wells were included as vehicle controls for normalization. After compound addition, cells were incubated for 96 hours at 37 degrees Celsius and 5% CO_2_. Following the incubation period, we added 10μL Cell Titer Glo reagent diluted 1:2 (Promega, Inc.) to each well and mixed. Plates were incubated for 5 min at room temperature, and luminescence was measured using an Envision multimodal plate reader (PerkinElmer, Inc.). Relative luminescence units were normalized to DMSO (vehicle) wells.

We quantified the combined effects of the 15 anti-cancer agents (**Supplementary Table 4**) against six different AML cell lines (FKH-1, HL60, TF-1, IDH2, Kasumi-1, K562; see **Supplementary Table 5**) using a high-throughput cell viability assay (CellTiter-Glo, Methods). Several of the tested drugs are clinically used to treat AML patients. ABT-199 is commonly used in combination with 5-azacitidine, and AG-221 is used in patients with IDH2 mutations^11, 12^. FKH-1 was isolated from a patient with Acute promyelocytic leukemia, HL60 was isolated from a patient who was presented with FAB M2 AML, an aggressive variant of AML. The Kasumi-1 cell line originates from an AML patient with t(8;21) translocation. The TF-1 cell line is derived from a patient with erythroleukemia, a subtype of AML. Meanwhile, IDH2 mutant of TF-1, which we will simply refer to as IDH2, is an isogenic cell line with the IDH2R140Q mutation, derived from the original TF-1 line^13^. These cell lines encompass high-risk (TP53 mutations in HL60, t(6;9) in FKH-1), low-risk (t(8;21) translocation in Kasumi-1 and t(15;17) translocation in HL60), and intermediate-risk (IDH mutations in TF-1 cells) genetic variants commonly observed in AML patients. In addition, we included the K562 erythroleukemia lymphoblast cell line that is derived from a patient diagnosed with chronic myelogenous leukemia.

### Data analysis

Every measurement was done in either duplicates or triplicates for every drug pair, drug concentration, and cell line. Raw absorbance values are normalized by using the median of at least 8 positive (only DMSO) and negative (inhibitor, 10mM BFA, brefeldin A, LC labs, Catalog B-8500) control wells on the same 384-plate. Equation 1 illustrates this step:

(1) $$v_{i,j}=\frac{y_{i,j}\;-\;n_{i}}{p_{i}\;-\;n_{i}}$$

where $v_{i,j}$ is normalized viability, $y_{i,j}$ is measured absolute absorbance intensities, $n_{i}$ is the median of negative controls, and $p_{i}$ is the median of positive controls. The median of all replicates of normalized viability measurements are used to create a single 8-by-8 or 11-by-11 matrix per drug pair per cell line, where each row represents increasing concentration gradient for drug A, and each column for drug B (Figure 1). The first row and the first column of these raw viability matrices represent single drug response curves (**Supplementary Figure 1**). The Bliss independence model was calculated by multiplying individual effects of each drug at each concentration interval and used as the reference baseline. A two-parameter sigmoid model was fitted for estimating IC_50_ for drug A at every drug B level in logarithmic scale with a base of 2. Equation 2 is used for estimating two parameters $b_{pos}$ representing the IC_50_ position, and $b_{shape}$ representing the steepness of the dose response:

(2) $$f\left(x,b_{pos},b_{shape}\right)=\frac{1}{1\;+\;e^{-b_{shape}*\left(x-b_{pos}\right)}}$$

where x is the base 2 logarithm of the drug concentration. For achieving continuity in the model fitting, zero drug level is approximated by a 2-fold decrease from the next lowest drug concentration level. Model optimization was done by solving the non-linear least squares problem using the Levenberg–Marquardt algorithm. Synergy or antagonism of a specific drug pair and cell line combination is quantified as follows:

(3) $$\Delta log2IC50_{A,B=b}=log\;2\frac{IC50_{A,B=b,observed}}{IC50_{A,B=b,expected}}$$

(4) $$\Delta log2IC50_{A,B,max}=max\left\{\Delta log2IC50_{A,B=0},\;\Delta log2IC50_{A,B=1},\ldots ,\Delta log2IC50_{A,B=7}\right\}$$

where A is the anchor drug A, and B is the library drug B, b is the index for increasing drug concentrations of the drug B. The direction of the synergy or antagonism is defined by the sign of the median IC_50_ change at all concentrations per drug pair cell line combination. For easier readability the synergy score is defined as $\Delta {log}_{2}\left({IC50}_{A,B,max}\right)$ and used as a metric for measuring synergism in the figures where synergy is positive, and antagonism is negative, and the base score of 1 represents a 2-fold change in IC_50_.

## Results

### Drug-drug interactions are mapped across six diverse myeloid leukemia cell lines

We examined synergistic and antagonistic interactions between drug pairs by utilizing two-dimensional (2-D, 8 by 8 grid) drug gradients in 384-well plates. Every 2-D gradient included single drug dose response curves (DRCs) for the measurement of two drugs in the first column and first row of each plate. We used a two-parameter sigmoid model to represent each single DRC (up to 14 curves per drug), obtain MIC (minimum inhibitory concentration) and IC_50_ (the inhibitory concentration required to reduce cell viability by half) values for each cell line (**Supplementary Figure 1** and **Supplementary Table 1**). In the layout depicted in Figure 1A, as the concentration of drug-B increases from left to right, the concentration of drug-A remains unchanged. Conversely, when the concentration of drug-A increases from bottom to top, the concentration of drug-B remains constant. At the starting point of each direction no drugs were added, and therefore at the bottom-left corner of each pattern the drug concentration is 0. For quality control, we incorporated both positive and negative controls on each plate to verify and gauge cell viability (further details are provided in the Methods section). By examining pairwise combinations of the 15 drugs on 6 distinct leukemia cell types, we determined the efficacy of 105 drug pairs at 3,167 possible combination regimes by performing 247,160 cell viability measurements.

We utilized the Bliss Independence Model as our neutral reference for measuring synergistic and antagonistic interactions between the drug pairs. As illustrated in Figure 1, for every pair of drugs, we examined cell viability at a constant dose of drug A while the concentration of drug B monotonously increased. First, we determined the anticipated cell viability under these conditions using the Bliss Independence Model (see Methods; local fits specific to each plate are employed). In Figure 1B, the expected cell viability is represented using black symbols at each concentration interval and a two-parameter sigmoid model shown as the black line. Subsequently, we contrasted the observed cell viability under these conditions to the expected cell viability. This analysis was repeated for each row (Figure 1A, black rectangle) with seven distinct constant concentrations of drug A.

For all cases, we determine the shifts in synergistic potency (Δlog2(IC_50_)) by computing the ratio between observed and predicted IC_50_ (S = log2(IC_50,observed_ / IC_50,predicted_)). Finally, to minimize discrepancies and effects of idiosyncratic measurements in our analysis, for each 8 by 8 gradient for a given drug pair applied to a given cell line, we classify each interaction either as synergistic or antagonistic by calculating the median synergy score (S, Methods) across all measured combination regimes. When the median value is positive, we label these interactions as synergistic and use the maximum achievable Δlog_2_(IC_50_) value as the synergy score (as depicted by red pixels in Figure 1D). Conversely, when the median value is negative, we label these interactions as antagonistic and use the minimum Δlog_2_(IC_50_) value as the synergy score (as depicted by blue pixels in Figure 1C). Therefore, we evaluate the synergistic potential of any drug pair against any cell line by the highest achievable synergistic or antagonistic effect across all tested combination ranges of those two drugs.

**Figure 2.**
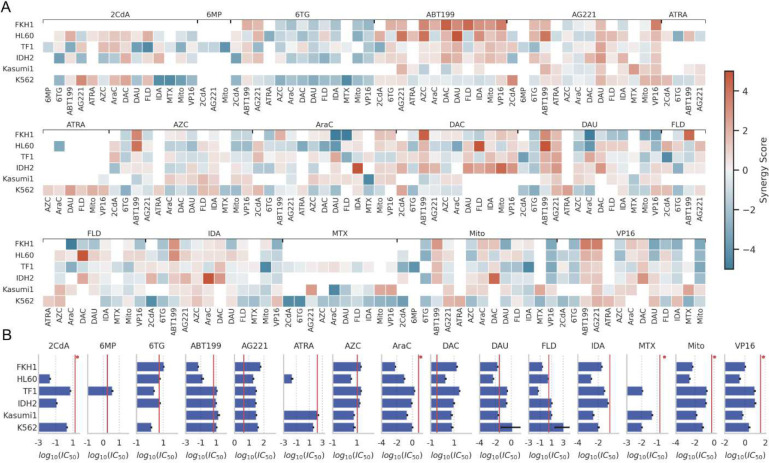
A. Synergistic and antagonistic potency of drug pairs are revealed. Synergy scores for every drug-drug-cell line pair with greater than two-fold IC_50_ gain in at least one cell line are shown on the heatmap. Drug pairs are labeled at the top and bottom x-axis while cell lines are annotated at y-axis. The full interaction heatmaps are presented in Supplementary Figure 2. **B. Single-drug efficacy values, i.e. IC**_**50,**_ **against the cell lines and clinically relevant drug concentrations.** Bars indicate mean values and error bars indicate standard deviation. Missing bars are due to very high IC_50_ values that were out of our experimental range or IC_50_ values that we could not unequivocally measure in our assay. Vertical red lines indicate maximum plasma levels for each drug based on previous clinical studies (Supplementary Table 6).

We provided synergy scores for all drug-drug-cell line pairs that resulted in a greater than 2-fold change (median of two or three replicates) in ΔIC_50_ against at least one cell line in Figure 2A, and the complete heatmap of the estimated synergy scores organized by drug pairs in both axes is provided as **Supplementary Figure 2**. Notably, the synergy scores between drug pairs we provide here only constitute a measure of how well the combination worked with reference to underlying single drug performance, rather than a measure of general efficacy. Therefore, we provide the efficacy levels of each drug for each cell line and clinically relevant plasma levels of these drugs (vertical red lines) in Figure 2B. Together they can be used to identify the drugs with the highest efficacy against a specific genetic background and also the highest synergistic gains through combination therapies. We note that in real-world scenarios, drug pharmacokinetics and pharmacodynamics do not maintain steady drug dosage regimens as we did in our *in vitro* measurements as much higher doses are used in clinical settings compared to what is used in our experiments.

The average synergy score across the 404 evaluated drug-drug cell line pairs was −0.005 ± 1.47 (mean ± standard deviation). This indicates that while the majority of drug interactions were Bliss independent (or additive), there was notable variability due to the large number of synergistic or antagonistic interactions. We identified 77 drug-drug-cell line pairs that had synergistic interactions, resulting in a reduction of IC_50_ values by at least two-fold (**Supplementary Table 3**). We identified 85 drug-drug-cell line pairs that displayed antagonistic interactions, where the combined action of the two drugs led to an increase in IC_50_ values by at least two-fold.

To ensure the experimental reproducibility we repeated measurements for a subset of the drug-drug cell line pairs (**Supplementary Figures 3 and 4**). For enhanced precision in these control experiments, we employed 11 by 11 drug gradients (in duplicates). We found that our measurements with 8 by 8 and 11 by 11 gradients were highly correlated (**Supplementary Figure 5**, Pearson’s correlation coefficient r = 0.67 with p = 6.7 × 10^−8^) ensuring experimental and computational reproducibility despite the significant alteration to the technical conditions of the experiment including tested drug concentration levels and intervals. Numerical values for all synergy scores and significant synergy scores can be found in **Supplementary Tables 4 and 5**, respectively.

### ABT-199 and AG-221 synergistically interact with several other drugs

In our study, every drug we examined exhibited synergistic interactions with at least five other drugs (Figure 2A). This implies that synergistic interactions are common and might not be attributed to any specific drugs. Notably, out of the significant synergistic interactions we observed, 47 out of 77 involved either ABT-199 or AG-221, two new generation cancer drugs. Both ABT-199 and AG-221 were involved in antagonistic interactions in a total of only 6 cases. ABT-199 particularly exhibited strong synergistic effects when combined with anthracyclines (DAU, IDA, MITO) and VP16. Similarly, AG-221 displayed strong synergy when used with these same drugs. Furthermore, these two drugs demonstrated synergistic interactions with one another across three distinct cell lines. This suggests that ABT-199 and AG-221 could potentially serve as enhancers of the effectiveness of many other AML treatments. Additionally, all six cell lines we tested had at least 8 synergistic drug-drug interactions with at least 2-fold IC_50_ reduction (FKH1: 14 pairs, HL60: 21 pairs, IDH2: 13 pairs, K562: 12 pairs, Kasumi1: 8 pairs, TF1: 9 pairs). These findings collectively indicate that synergistic drug pairs for AML treatment are likely to be identified across various genetic contexts. We note that ABT-199 and AZC are currently used to treat older AML patients. However, in our study, we found that while FKH1 and HL60 cell lines demonstrate significant synergy with this combination, Kasumi1 and K562 do not respond as effectively. This suggests that clinical outcomes for the ABT-199 and AZC combination can vary depending on the genotypic background of the AML.

### 6-Thioguanine interacts antagonistically with several other drugs.

Antagonistic interactions between drugs were also widespread across all cell lines and every drug we tested (Figure 2A). Notably, of these interactions, 29 out of 85 involved 6-Thioguanine (6-TG), a purine analog. This suggests that although 6-TG has been used for acute lymphoblastic leukemia treatment, it may antagonize the effects of many treatments used for AML. Interestingly, the two drugs, ABT-199 and AG-221, which were highlighted for their strong general synergistic potential, evaded the strong general antagonistic effect of 6-TG.

### Combined effect of cytarabine (AraC) and daunorubicin (DAU) is generally additive

The “3 + 7 regimen” (3 days of daunorubicin + 7 days of cytarabine) established in the 1970s became the standard AML treatment^14^. CPX-351, a nanoliposome encapsulating cytarabine and daunorubicin in a 5:1 molar ratio, has been shown to significantly improve survival for some patient groups, increase complete remission rates, and facilitate more successful stem cell transplantations (SCT), extending post-SCT survival. This led to its FDA approval as the primary treatment for secondary AML. However, in our experiments, this drug pair did not exhibit pronounced synergistic interactions (Figure 2). Their combined effects displayed weak synergy in TF-1, Kasumi-1, and IDH2 cell lines but were weakly antagonistic in FKH-1, HL60, and K562. As we mentioned before, our assay is conducted at fixed concentrations of these compounds and cannot replicate the drug dose fluctuations in patients. In view of the critical impact and characteristics of the AraC and DAU combination, we examined how the effect varies in a dose-dependent manner on each cell line and found that drugs acted independently except in the case of FKH-1, for which we observed moderate synergy (**Supplementary Figure 8**).

### Cytarabine use in combination with alternative anthracyclines

Combining cytarabine with alternative anthracyclines such as mitoxantrone and idarubicin and establishing their optimal doses has been the subject of several randomized trials^15^. Both drugs are known to be active against leukemia cell lines that are resistant to daunorubicin^16^. Whether these drugs exert a differential activity on normal hematopoietic stem cells remains unclear. However, the comparable toxicity of the three drugs in combination with conventional-dose cytarabine and etoposide during induction does not necessarily imply that the same doses of the drugs have equivalent effects with the combination of intermediate-dose cytarabine during post remission chemotherapy. In our study, we observed significant antagonism between cytarabine and daunorubicin in FKH-1 and HL60 cell lines which were partially or fully reversed when combined with idarubicin and mitoxantrone (Figure 2). The reverse of this shift in synergistic interaction is observed in the TF1 cell line, corroborating the hypothesis that, in clinical cases for which the typical AraC-DAU combination is not effective, using a different anthracycline to replace daunorubicin could prove beneficial.

### Drug-drug interactions are not conserved across all AML genotypes

**Figure 3.**
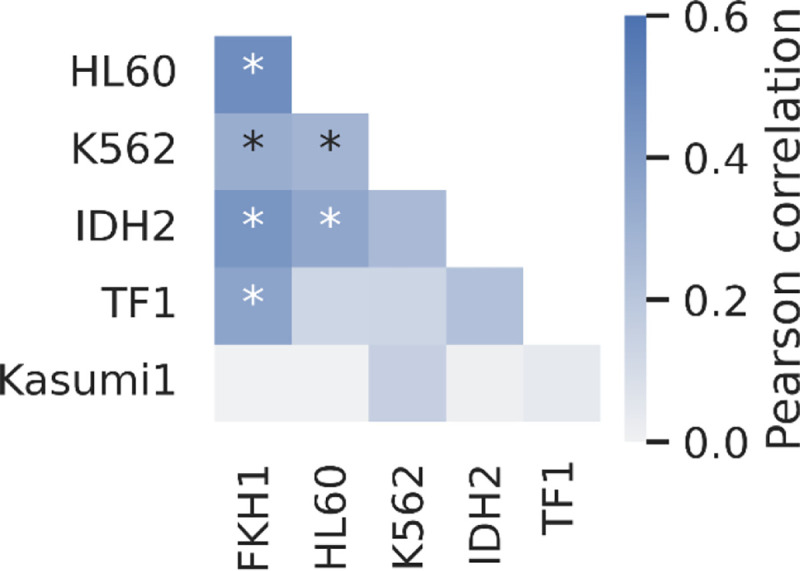
Synergistic and antagonistic drug-drug interactions are weakly correlated across cell lines. Pearson correlation between synergy scores in all cell lines are compared pairwise. Pearson correlation coefficient is colored with blue as indicated by the color bar, and stars indicate where p-value is less than 0.05.

We assessed the conservation of drug-drug interactions across various cell lines by analyzing the correlation between synergy scores in these cell lines (Figure 3). The average correlation across the cell lines was 0.198 ± 0.175 (mean ± standard deviation). Considering all varying technical conditions of the experiments including the study personnel, drug concentration intervals, culturing of the cell lines, experiment dates and randomized well selections across two experiments with duplicates, our predicted synergy scores produce a Pearson correlation of 0.67 **(Supplementary Figure 5),** this indicates that the conservation of drug-drug interactions across different genetic backgrounds was weak. Among the cell lines we tested, the synergy scores from the Kasumi-1 cell line showed the weakest correlation with the synergy scores obtained from other cell lines, with an average of −0.001 ± 0.099 **(Supplementary Figure 6)**. In contrast, the strongest correlation was observed between the HL60 and FKH1 cell lines, with a Pearson correlation of 0.47.

### One mutation can significantly alter single-drug efficacy and drug-drug interactions

**Figure 4.**
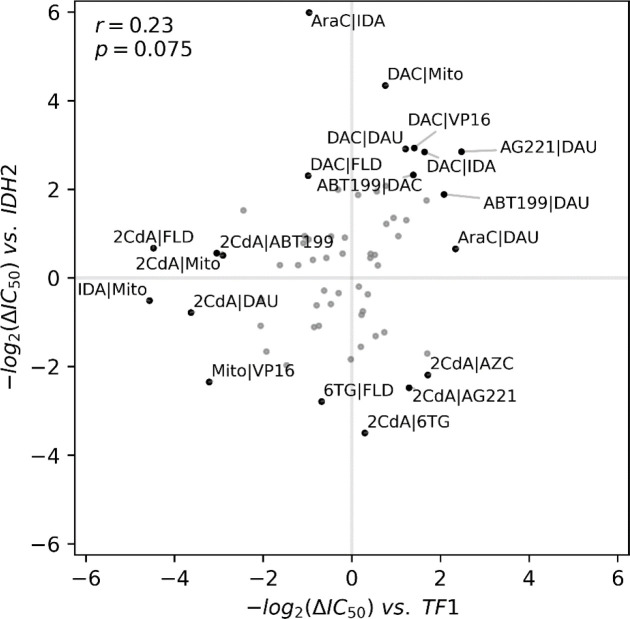
An IDH2 mutation significantly alters the drug-drug interaction landscape in TF1. Measured synergy scores in cell lines IDH2 and TF1 are plotted. Pearson correlation and corresponding p-value is provided at the top left.

Mapping variations in drug-drug interactions to the genotypes of cell lines is a complex task and beyond the scope of this study. Yet, to explore the sensitivity of this clinically important phenotype to genetic variations, we contrasted the synergy scores for drug pairs between the TF-1 and IDH2 cell lines. The IDH2 cell line is an isogenic derivative of the original TF-1 and carries only the IDH2-R140Q mutation^13^. IDH2 is an enzyme which catalyzes α-ketoglutarate, and when mutated is known to induce changes in methylation^17^. Due to the small genetic distance between these two cell lines compared to other cells, one would expect that drug-drug interactions in IDH2 and TF-1 would be similar. However, intriguingly, the correlation of synergy scores between the TF-1 and IDH2 cell lines was very low (r= 0.23, Figure 4), indicating that even a single mutation can potentially shift the outcomes of combined drug treatments. Several of the additive interactions in TF-1 shifted to antagonism or synergy in the IDH2 cell line. Most strikingly, the synergistic interaction between AG-221 and 2CdA in TF-1 became antagonistic in IDH2 cells. Figure 4 also shows examples of conserved antagonism (for instance, between mitomycin and VP16) and synergy (such as ABT-199 and DAC) in both IDH2 and TF-1 cell lines. Nonetheless, there are numerous drug-drug interactions that displayed synergy or antagonism in the TF-1 cell line but showed additive interactions in the IDH2 cell line.

## Discussion

In our analysis of pairwise interactions for 15 commonly used anticancer drugs in six diverse cell lines originated from acute myeloid leukemia, we identified numerous synergistic and antagonistic interactions between drug pairs. Notably, AG-221 and ABT-199, recent additions to the cancer treatment arsenal, accounted for the highest counts of synergistic interactions and the fewest counts of antagonistic interactions. This finding underscores the potential of both AG-221 and ABT-199 to act as synergistic agents in multi-drug therapies. This finding is consistent with the clinical effectiveness of ABT-199 against AML when combined with a variety of other therapies such as azacytidine^18^, low dose cytarabine^19^, and combination chemotherapy^20^. Indeed, ABT-199 combined with AZC has emerged as a standard treatment for elderly AML patients^21^, and trials of ABT-199 with other regimens and combinations continue^22^. Although ABT-199 and AG-221, both individually and in combination, show promise for AML treatment, they have yet to become definitive solutions.

Although our study is conducted using cell lines and its outcomes cannot be directly applied to clinical settings, our findings align with a recent clinical study by Zeng and colleagues, which also showed that individuals and cell lines with different genetic backgrounds respond variably to AML treatment^23^. Their analysis of over 1,000 AML patient samples revealed significant genetic heterogeneity and differences between mature and immature cancer cells. They reported that different genetic and biological subgroups of AML had varying responses to both single and combination therapies. They also found that patients with mature cancer cells had lower survival rates with current treatment regimens. Additionally, venetoclax (ABT-199) was found to be more effective against primitive cell lines, whereas monocyte-like cells exhibited resistance to the treatment.

There are ongoing trials examining the potential of ABT-199 in combination with anthracyclines and intensive regimens, including various doses and durations of ABT-199 with ARAC+DAU^2^. Ongoing studies assessing the efficacy of ABT-199 also include its use with FLAG-IDA (FLU+ARAC+IDA)^24, 25^; with CLIA (2CDA, IDA, ARAC)^25^ and with only 2CDA^26^. Enasidenib (AG-221) on the other hand is currently used in clinical settings to treat AML patients with IDH2 mutations, administered with ARAC+DAU combinations during induction or consolidation^27^, or with AZC^28^. A previous study on the use of the AG-221-DAU combination suggested that AG-221 synergizes with daunorubicin by inhibiting AKR1C3 enzyme and preventing ABC transporters’ activities, thereby sensitizing cancer cells against drug molecules^29^. These findings also align with our results, in which we show that AG-221 synergistically interacts with several drugs, and particularly, AG-221 works synergistically with both DAU and VP16 in all cell lines.

We observed both synergistic and antagonistic drug-drug interactions in all cell lines we tested. However, these interactions were not universally conserved across different cell lines, as depicted in Figure 3. Furthermore, it was not possible to attribute synergy or antagonism in combinatorial drug interactions to a particular drug or group of drugs, since every compound we tested was involved in several synergistic or antagonistic interactions as summarized in Figure 2. This variability suggests that the efficacy of combined therapies is closely linked to the genetic context of the disease^30^. As we demonstrated in Figure 4, even a single mutation in the TF1 cell line can profoundly alter drug-drug interactions, even converting synergistic ones to antagonistic, as seen with AG-221 and 2CdA.

In conclusion, both synergistic and antagonistic drug interactions are prevalent among anti-cancer drugs in various genetic backgrounds. Recognizing and accounting for these interactions constitutes a largely untapped resource for designing effective combination therapies for AML treatment. The findings and conclusions of this study are derived from high-throughput *in vitro* cell viability measurements, and we make no claims regarding the clinical efficacy or relevance of drug pairs. Although most of the drug regimens we use in our study are within the clinically relevant drug concentration windows in plasma (Figure 2B), it remains uncertain whether our observations will hold true in preclinical and clinical settings. Nevertheless, our research can offer valuable insights and serve as a foundation for clinical studies, potentially enhancing the efficacy of chemotherapies and, in the long run, improving patient treatment outcomes.
